# Drivers of Artificial Intelligence (AI) Adoption in Supporting Chronic Disease Management in Primary Care: A Scoping Review

**DOI:** 10.3390/nursrep16090315

**Published:** 2026-09-02

**Authors:** Tao Wang, Jing-Yu (Benjamin) Tan, Mengyuan Li, Haiying (Emily) Wang, Sita Sharma, Daniel Terry

**Affiliations:** 1 School of Nursing and Midwifery, Edith Cowan University, Joondalup, WA 6027, Australia; alison.wang@ecu.edu.au; 2 School of Nursing and Midwifery, University of Southern Queensland, Ipswich, QLD 4305, Australia; benjamin.tan@nd.edu.au (J.-Y.T.); mengyuan.li@unisq.edu.au (M.L.); emily.wang@unisq.edu.au (H.W.); sita.sharma@unisq.edu.au (S.S.); 3 Institute for Health Research, University of Southern Queensland, Springfield, QLD 4350, Australia; 4 School of Nursing and Midwifery, The University of Notre Dame Australia, Fremantle, WA 6160, Australia; 5 Institute of Health and Wellbeing, Federation University Australia, Ballarat, VIC 3353, Australia; 6 School of Nursing, Shiga University of Medical Science, Otsu 520-2192, Shiga, Japan

**Keywords:** artificial intelligence, primary care, chronic disease management, digital health, scoping review, nursing

## Abstract

**Background/Objectives**: Chronic diseases place sustained pressure on primary care systems worldwide. While artificial intelligence (AI) shows promise for supporting chronic disease management in primary care, the factors influencing its adoption and implementation have not been comprehensively addressed within the specific context of primary care chronic disease management. The aim of the scoping review was to identify the key drivers that influence AI adoption in primary care settings for chronic disease management. **Methods**: This scoping review was conducted following the PRISMA ScR guidelines. A comprehensive search of seven databases was conducted between 14 April and 14 May 2025, with an updated search on 27 February 2026 using the same search strategy and eligibility criteria. Eligible studies examined the adoption or implementation of AI for chronic disease management in primary care and were published in English from 2010 to 2026. Data were charted using a standardised extraction tool and synthesised narratively. The protocol for this scoping review was registered with INPLASY. **Results:** Twenty-two studies were included, identifying five domains influencing AI adoption. Technical factors included data quality, interoperability, and algorithm transparency. Human factors related to clinician trust, workload, digital literacy, and preferences for hybrid human–AI care. Legal and ethical considerations centred on privacy, accountability, fairness, and independent validation. Organisational factors involved leadership, workflow integration, and resourcing, while geographical and cultural contexts shaped readiness, acceptability, and equity. **Conclusions:** AI adoption in chronic disease management within primary care is shaped by multi-level, context-dependent factors. The findings suggest that implementation may require coordinated strategies encompassing robust data governance and interoperability, clear regulatory frameworks, workforce capability development, workflow-aligned hybrid models with appropriate human oversight, and sustainable resourcing and reimbursement. Future research should extend beyond technical performance to evaluate acceptability, safety, workload, equity, and longer-term organisational impacts.

## 1. Introduction

Chronic diseases, often grouped as noncommunicable diseases (NCDs) such as cardiovascular disease, diabetes, chronic respiratory disease, and cancer, remain the leading cause of morbidity and mortality worldwide [1]. The World Health Organization (WHO) estimates that NCDs caused at least 43 million deaths in 2021, representing around three-quarters of non-pandemic-related deaths globally [2]. Primary care is widely recognised as the cornerstone of chronic disease prevention and long-term management, providing accessible, continuous, and coordinated care across the disease trajectory [3]. Evidence from primary and community care interventions indicates that chronic disease management approaches, particularly those aligned with elements of the Chronic Care Model and self-management support, are commonly associated with improvements in care processes and patient outcomes [4]. However, increasing service demand and workforce capacity constraints, alongside fragmented (often disease-focused) systems of care and the rising prevalence of multimorbidity, continue to challenge primary care’s ability to deliver timely, person-centred and sustainable chronic disease management at scale, highlighting the need for context-specific approaches to artificial intelligence (AI) implementation in primary care settings [5,6,7].

AI, including machine learning, natural language processing, and other data-driven techniques, has been increasingly applied across healthcare to support more efficient healthcare practices, such as risk stratification, symptom detection and assessment [8], clinical decision-making [9,10], and patient self-management [11]. Within the context of primary care, AI has been explored as a potentially transformative capability for enhancing diagnostic and prognostic accuracy [12,13], prioritising high-risk patients, tailoring treatment plans [14], and enabling more proactive, personalised chronic disease monitoring and support [15]. In chronic disease management, AI-enabled tools are also recognised as a promising way to synthesise complex longitudinal data (e.g., electronic health records, wearable and home monitoring data, and patient-reported outcomes) to support more efficient symptom detection and monitoring, evidence-informed actions, reduce clinical burden, and strengthen continuity of care [12,16,17]. Nevertheless, the extent to which these potential benefits can be realised in primary care for patients with chronic disease is likely to depend on safe real-world integration and alignment with prevailing care models, workflows, and the needs of patients and clinicians. As such, AI adoption is not only a technical challenge but also an implementation challenge shaped by organisational, social, and regulatory conditions. For instance, adoption and sustained use may be influenced by how well AI tools fit routine workflows and usability requirements, as well as trust and governance arrangements [18,19]. Practical feasibility may also hinge on data quality and interoperability [19], alongside medico-legal accountability and regulatory/liability considerations [18,20]. Equity and patient safety are additional prerequisites for acceptable and responsible implementation [19,20].

Several existing reviews have synthesised factors of AI adoption across healthcare settings. For example, Hassan et al. [19] identified factors such as trust, governance, and regulatory considerations as central to AI adoption across healthcare contexts. Assadullah [21] similarly emphasised barriers including technological limitations, lack of expertise, and resistance to change among healthcare professionals. Chomutare et al. [22] applied the Consolidated Framework for Implementation Research (CFIR) to map the key drivers reported in 19 empirical studies, offering a theory-informed view of implementation determinants. Meanwhile, Jacob et al. [23] proposed the AI for IMPACTS framework, arguing that evaluation should extend beyond technical performance to capture longer-term social, organisational, and real-world impacts.

While these reviews provide valuable cross-cutting insights into AI adoption across healthcare contexts, they remain largely system-level and do not account for the distinctive clinical, organisational, and relational characteristics of primary care chronic disease management. Factors such as continuity of care, multimorbidity, time-constrained consultations, and community-based service delivery create implementation conditions that differ substantially from those in other healthcare settings. Consequently, there remains a need for a focused synthesis of the factors influencing AI adoption within this specific context.

This review extends prior work by applying an implementation science lens, guided by the CFIR, to systematically map multi-level drivers of AI adoption across technical, human, organisational, ethical, and contextual domains. In doing so, the aim is to identify the key drivers that influence AI adoption in primary care settings for chronic disease management. By examining implementation drivers across multiple domains, it seeks to provide a more nuanced understanding of how AI can be integrated into routine primary care workflows while supporting uptake, sustainability, equity, and patient-centred care. Accordingly, the contribution of this review is threefold: (1) it bridges the gap between general AI-in-healthcare adoption literature and the specific realities of chronic disease management in primary care; (2) it advances conceptual understanding by using CFIR as an interpretive implementation framework to contextualise implementation drivers in this context; and (3) it offers practical, context-sensitive insights to inform policy, practice, and future implementation research.

## 2. Methods

### 2.1. Study Design and Registration

A scoping review design was adopted to identify and synthesise the key drivers that influence the adoption of AI in primary care settings for chronic disease management. This scoping review is reported by following the PRISMA for Scoping Reviews (PRISMA-ScR) guideline [24]. The objectives, analysis methods, and inclusion and exclusion criteria were developed and documented as outlined by Tricco et al. [24] to ensure accurate and complete reporting of findings. In addition, the theoretical framework guiding the review was the CFIR, which is a multi-domain framework designed to assess factors that influence the implementation of new interventions in healthcare and other settings. In this review, CFIR was used as an interpretive implementation framework rather than as a prescriptive coding structure. The updated CFIR comprises five domains: Innovation, Outer Setting, Inner Setting, Individuals, and Implementation Process [22,25]. A hybrid inductive-deductive approach was used to enable factors specific to AI adoption and the primary care context to emerge from the included evidence and then be examined in relation to established CFIR constructs. CFIR is well-suited to examining the drivers of AI adoption in primary care for chronic disease management [22,25]. The protocol for this scoping review was registered with INPLASY (DOI: 10.37766/inplasy2026.1.0096). AI or AI-assisted tools were not used in drafting any aspect of this manuscript; except for the development of a figure in the Section 3.

### 2.2. Eligibility Criteria

Studies were included if they met the following criteria: (1) focused on the adoption or implementation of AI technologies for chronic disease management; (2) identified drivers that impact AI adoption; (3) were conducted in primary care settings such as general practices, family medicine clinics, or community health centres; (4) employed empirical research methods, including qualitative, quantitative, or mixed-methods designs; (5) published in English; and (6) released between January 2010 and February 2026, to reflect the rapid advancements in AI technologies. CFIR was not used as a study selection criterion; rather, it was applied during data extraction and synthesis to categorise and interpret factors influencing AI adoption identified across the included studies. Validation, predictive modelling, feasibility, and observational studies were eligible where they reported factors likely to influence implementation or adoption, including issues related to data quality, workflow integration, usability, transparency, deployment requirements, or stakeholder acceptability.

Studies were excluded if they (1) were conducted exclusively in secondary or tertiary care settings; (2) focused on acute conditions or short-term interventions; (3) addressed digital health interventions without an AI component; or (4) lacked empirical research data, such as opinion pieces or commentaries.

### 2.3. Information Sources

A comprehensive literature search was conducted between 14 April and 14 May 2025 and updated on 27 February 2026 using the same search strategy and eligibility criteria to identify newly published studies. The search was limited to peer-reviewed literature, as the review aimed to synthesise empirical evidence from scholarly publications; therefore, grey literature (e.g., policy reports, organisational documents, and technical reports) was not included.

The following electronic databases were searched: MEDLINE (via PubMed), EMBASE, CINAHL, PsycINFO, Scopus, IEEE Xplore, and the Cochrane Library. Search strategies were developed using a combination of controlled vocabulary (where available) and free-text keywords and were adapted to the indexing structure and search functionality of each database. Depending on database capabilities, searches were conducted across subject headings, titles, abstracts, and author keywords, with appropriate field restrictions applied to optimise retrieval. The complete search strategies for each database are provided in Appendix A.

For Scopus, the final search was limited to title and abstract fields. Preliminary searches that included broader field options retrieved a substantial number of records in which the search terms were only incidental and not central to the study objectives, resulting in low search precision. Restricting the search to title and abstract fields improved the relevance of retrieved records by capturing studies in which the key concepts constituted the primary focus while maintaining a comprehensive and systematic approach to study identification. This restriction was not considered likely to materially affect study identification because searches were conducted across multiple complementary databases using broader indexing fields where available, and reference list screening was also undertaken to identify potentially missed studies.

### 2.4. Search Strategy

Key search terms included are as follows: ((“primary care” OR “general practice” OR “family medicine” OR “community health”) AND (“artificial intelligence” OR “AI” OR “machine learning” OR “deep learning”) AND (“adoption” OR “implementation” OR “barriers” OR “facilitators” OR “challenges” OR “enablers”) AND (“chronic disease” OR “chronic illness” OR “long-term conditions” OR “diabetes” OR “hypertension” OR “COPD” OR “cancer”)). Word variations and suffixes were also included. Detailed search strategies of all used databases are presented in Table A1, Table A2, Table A3, Table A4, Table A5, Table A6 and Table A7 (Appendix A). In addition, hand searching and reviewing of reference lists of identified studies was undertaken to uncover any extra studies that may not have been captured by the literature search.

### 2.5. Study Screening

Retrieved articles were exported to and managed using EndNote (version 21) and were initially screened by two reviewers (DT and MYL) after duplicates were removed. All studies were initially screened based on titles, keywords, and abstracts to exclude irrelevant articles. In the second round, the remaining full-text articles were then assessed independently by two reviewers (DT and MYL) and judged against the inclusion and exclusion criteria. Each study was classified as ‘include’, ‘exclude’ or ‘unsure’ in the review. Any discrepancies between the two reviewers were resolved by a third reviewer (EW) until consensus was achieved. To ensure consistency, eligibility criteria were discussed and clarified among reviewers prior to screening and refined iteratively through regular discussion during the screening process.

### 2.6. Methodological Quality Assessment

Methodological quality appraisal was not conducted, consistent with scoping review guidance that critical appraisal is optional and should align with the review purpose [24]. As this review mapped drivers of AI adoption in primary care (not effectiveness), studies were charted descriptively and analysed thematically without quality-based exclusions. This approach aligns with scoping review methodology, which prioritises mapping the breadth and nature of available evidence rather than evaluating study quality or excluding studies based on methodological rigour.

### 2.7. Data Extraction and Analysis

Data were extracted using a standardised approach. For each included study, details on author/year, country, study design, population/sample, AI intervention/exposure, outcomes, and key findings were charted. Data relating to factors influencing AI adoption or implementation, including reported barriers, facilitators, enablers, concerns, contextual influences, and relevant study limitations, were extracted. This was undertaken by two reviewers (DT and MYL) using the words and meanings reported in the original studies.

Textual data were synthesised using a narrative synthesis approach [26] with two linked stages. First, an inductive synthesis was undertaken to preserve determinants specific to AI adoption and the primary care context. The included articles were read iteratively to identify key statements and meanings, which were compared across studies and coded according to their underlying meaning. Conceptually similar codes were then grouped and refined into five empirically derived overarching domains aligned with the review aim, including technical factors, human factors, legal and ethical factors, organisational factors, and cultural/rural-specific factors. These domains were therefore derived from the included evidence rather than predetermined from CFIR.

Where findings reflected similar meanings, they were aggregated to confirm common patterns [26]. Where findings were thematically diverse, a configurative approach was used to integrate complementary or contrasting insights to deepen interpretation and develop a more nuanced understanding [27]. Following development of the inductive synthesis, the identified factors were deductively mapped to relevant domains and constructs within the updated CFIR [25]. Mapping was undertaken based on the conceptual meaning and underlying intent of each factor rather than the specific terminology used within individual studies. Where factors were relevant to multiple CFIR constructs, a primary mapping was assigned according to best conceptual fit, while secondary alignments were noted where appropriate. Factors that were not adequately represented within existing CFIR constructs were retained within the inductively derived domains to preserve conceptual integrity and avoid forcing findings into predefined categories. This inductive-first approach was adopted as many implementation determinants identified in the AI literature span multiple CFIR constructs and may not be adequately captured by a single predefined domain, particularly factors relating to technical performance, data governance, and cultural context. The CFIR was therefore used as an organising framework to contextualise and interpret findings rather than as a restrictive analytical structure. Given heterogeneity in study aims, methods, and outcomes, meta-analysis was neither appropriate nor feasible.

## 3. Results

### 3.1. Study Selection and Characteristics of the Included Studies

**Figure 1:**
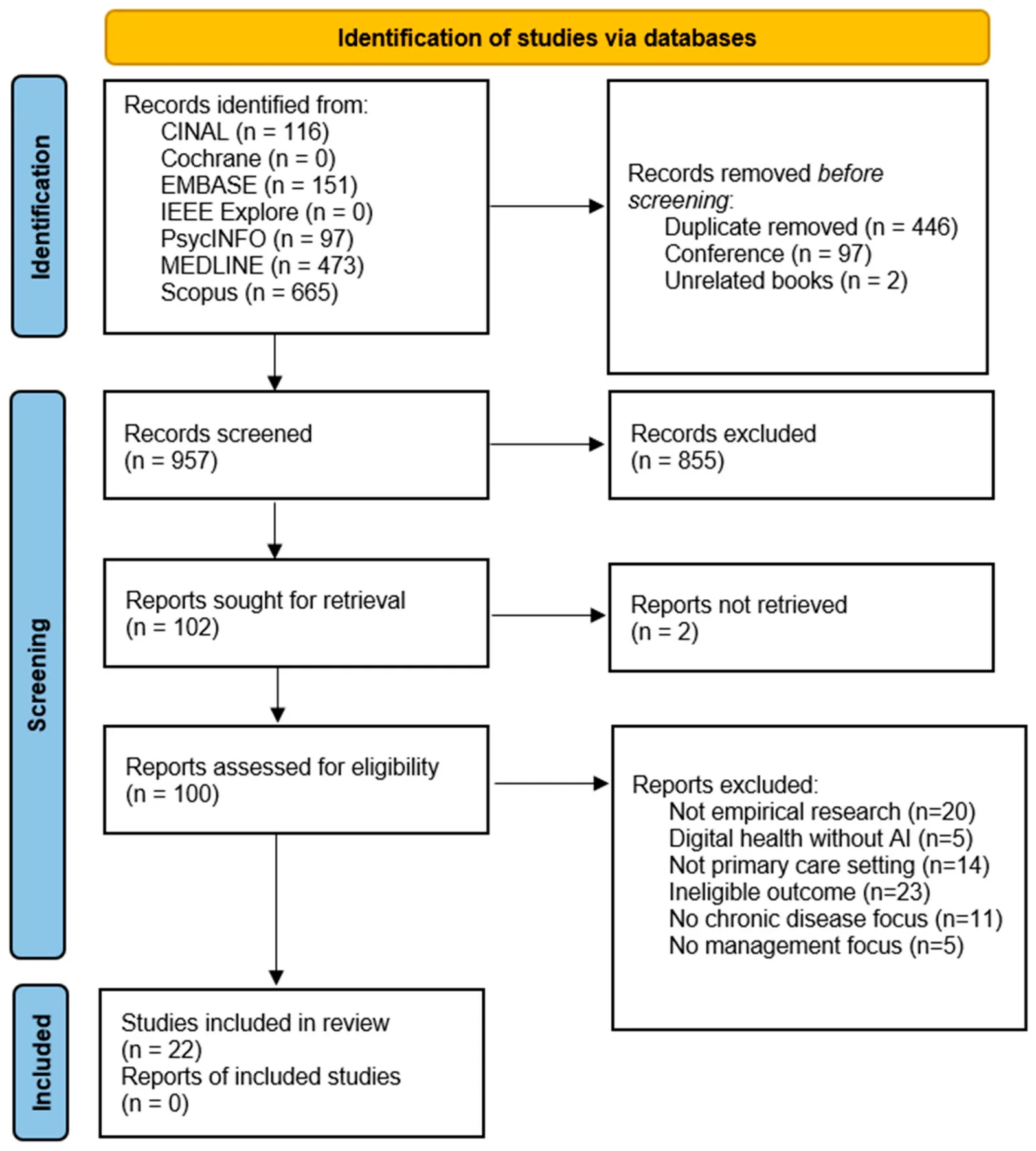
PRISMA ScR flow diagram.

The literature search yielded 1502 potentially relevant publications, and after removing duplicates (*n* = 446) and articles that did not meet the inclusion criteria (*n* = 99), *n* = 957 potential articles were identified. After title and abstract screening and following the exclusion of records for which the full text could not be retrieved, 100 full-text articles were assessed for eligibility. Of these, 78 articles were excluded following full-text review. A total of 22 studies were agreed upon for inclusion in this scoping review (Figure 1). Overall, the final group of publications included eight qualitative [28,29,30,31,32,33,34,35], five cross-sectional [36,37,38,39,40], one case–control [41], two validation [42,43], and two implementation studies [44,45]. In addition, one cohort [46] and three observational studies [47,48,49] were also identified among the articles. This concentration suggests the evidence is weighted towards screening-focused AI applications, and findings should therefore be interpreted with consideration that other areas of chronic disease management (e.g., multimorbidity care, medication management, and self-management support) are comparatively underrepresented.

Among the identified studies, five were conducted in the UK [30,33,34,35,44], four in the US [36,41,47,48], three in India [39,40,49], two in Germany [32,37], with one study conducted in Australia [45], Brazil [42], Canada [46], Denmark [31], the Netherlands [29], Saudi Arabia [38], and Singapore [28]. In addition, one study was conducted across two countries, which encompassed the US and Thailand [43]. Of the 22 articles, ten focused on diabetic retinopathy screening [31,32,36,37,40,43,45,46,47,48] and two on diabetes management [28,47], while the remainder examined chronic wound dressings [42], liver function investigations [44], skin cancer care [29], geriatric telemedicine [39], assistance with spirometry [30], or general family medicine, including medication management [33,34,35,38]. Lastly, one article [41] focused on using AI for eye disease risk prediction, which included diabetic retinopathy among chronic eye diseases. To enhance clarity, a concise summary of key study characteristics is presented in Table 1, with full details provided in Supplementary Table S1 (Supplementary File S1).

### 3.2. Identified Drivers Impacting AI Adoption for Chronic Disease Management in Primary Care

**Figure 2:**
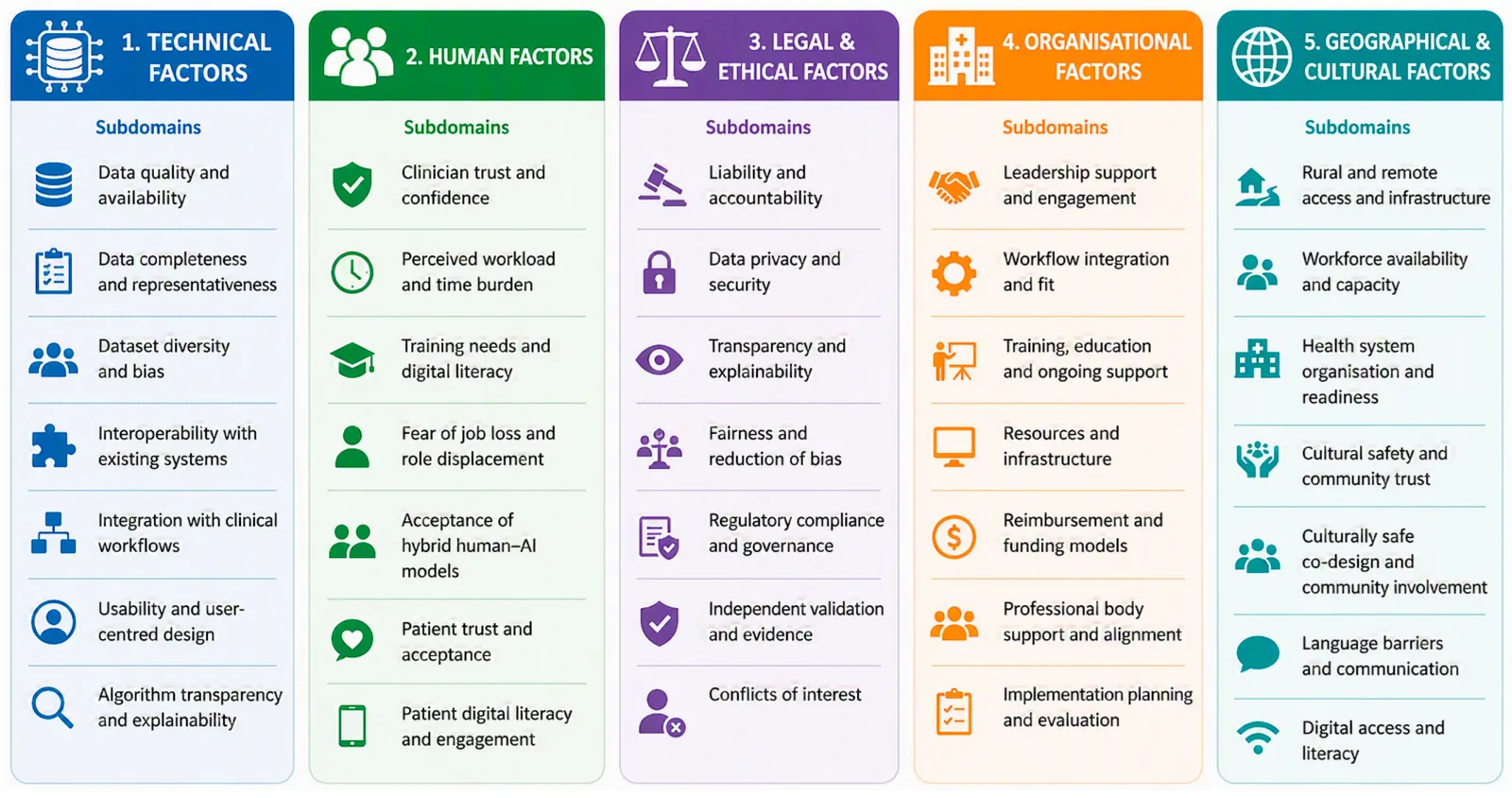
Key drivers of AI adoption in primary care settings.

Inductive synthesis identified five overarching categories of drivers that impact AI adoption for chronic disease management in primary care, which included technical barriers (e.g., data quality and system interoperability), human factors (e.g., clinician trust and patient preferences), legal and ethical challenges (e.g., liability and data privacy), organisational factors (e.g., leadership support and workflow integration), and geographical and cultural contexts. Table 2 presents the distribution of the five identified driver categories across the included studies. These categories represent the empirically derived structure of the included evidence rather than the five CFIR domains. Subsequent deductive mapping showed that individual factors spanned multiple constructs within the updated CFIR; the relationships between the five empirically derived domains and relevant CFIR constructs are summarised in Table 3 and Figure 2. Each factor is presented below:

#### 3.2.1. Technical Factors

Technical factors were a major concern in the adoption of AI for chronic disease management in primary care. A recurring issue was the limited availability and quality of data required to train and validate AI models. Young et al. [41] highlight the absence of critical imaging data, such as Optical Coherence Tomography data, to confirm Clinically Significant Macular Oedema, which undermined the robustness of their AI screening tool [36,43]. Conversely, another study [33] highlighted that there remains an overreliance on structured Electronic Health Records data, which may not capture the full clinical picture, thereby limiting the generalisability of AI models across diverse patient populations. Similarly, Bhambhwani et al. [46] raised concerns about the lack of diversity in training datasets, suggesting this could lead to biased outputs and reduced diagnostic accuracy for underrepresented groups.

Integration and usability also pose significant technical hurdles, as Wewetzer et al. [37] reported that high device costs and poor interoperability with existing practice systems hinder the seamless adoption of AI tools. Data security concerns further complicate implementation, particularly in settings where digital infrastructure or internet reliability is limited [32,39,40,49]. Sangers et al. [29] emphasised the complexity of AI algorithms and the lack of integration with clinical context, which was suggested to erode clinician confidence and limit clinical utility. Studies from da Silva Lima Roque et al. [42] and Yoon et al. [28] both highlighted the importance of usability testing, particularly for tools like chatbots and mobile apps, which need to be intuitive for both clinicians and patients. These studies collectively suggested that without addressing these technical limitations, AI tools may struggle to gain traction in real-world primary care environments.

#### 3.2.2. Human Factors

While technical factors such as interoperability, algorithmic complexity, and usability remain critical, the adoption and effectiveness of AI in primary care are fundamentally shaped by human factors, including how clinicians and patients perceive, trust, and are ready to use these tools. These findings are derived from a mix of clinician-only studies, patient-focused studies, and mixed stakeholder samples. Clinicians, particularly in the qualitative studies of general practitioners and primary care providers, generally express cautious optimism about AI’s potential to enhance diagnostic accuracy and streamline triage, particularly in chronic disease contexts such as diabetic retinopathy, provided that solutions integrate seamlessly with existing systems and minimise additional workload [36,41,45]. Persistent concerns remain regarding the reliability and transparency of AI systems [34,35]. Workforce implications, such as de-skilling and potential role displacement, were also noted, along with concerns that it may undermine the therapeutic clinician–patient relationship [34]. These issues appeared to be reported more frequently by younger clinicians. Participants further highlighted the time and training burden required to use AI effectively. It was suggested that trust in AI increased when tools were validated and endorsed by reputable institutions. General practitioners, however, were often more cautious than specialists due to their broader scope of practice and concerns about safety and liability [29,31,32,37,47].

From the patient perspective, based on studies involving patients with diabetes, older adults, and community-based populations, satisfaction is high when AI improves convenience, reduces wait times, and brings services closer to home, such as AI-enabled diabetic retinopathy screening and mobile health apps. However, many, particularly older adults and those with low digital literacy and limited confidence, indicate they value human empathy, prefer hybrid models with clinician oversight, and encounter digital literacy barriers [39,40,48]. Trust increases with perceived accuracy, clinician endorsement, and clear, understandable guidance, while privacy and decision transparency remain salient concerns [38,41,42,46,48]. Collectively, these findings suggest a need for trustworthy, usable, workflow-aligned AI with explicit human oversight, education, and reassurance to support safe integration into primary care [28,29,41].

#### 3.2.3. Legal and Ethical Factors

Legal and ethical factors were also noted to be central to the responsible use of AI in healthcare, particularly in primary care settings where patient trust and safety are paramount. One of the most pressing issues noted was liability in terms of ‘who’ is responsible when an AI system makes an incorrect recommendation or diagnosis [33,35]. Sangers et al. [29] raise this concern alongside other broader issues of fairness and health equity, warning that without proper oversight, AI could exacerbate existing disparities in healthcare. Wewetzer et al. [37] also highlight the tension between innovation and privacy, noting that commercial interests in AI development may conflict with the inherent ethical obligation to protect both patients and their data. Issues of data privacy and cybersecurity similarly emerged as central barriers to trust in AI-enabled telemedicine [39].

Transparency and autonomy were also noted as additional ethical dimensions that needed to be addressed. For example, Young et al. [41] were funded by an AI developer and lacked Optical Coherence Tomography confirmation for diabetic retinopathy, raising concerns regarding potential biases and the need for independent validation. Yoon et al. [28] raised ethical concerns about how AI makes decisions and how that impacts the ability of patients to make informed choices. They stressed the need for AI tools to be understandable so that both doctors and patients can see how and why a decision was made. Conversely, da Silva Lima Roque et al. [42], with their focus on chatbot usability, suggested a need for safeguards to ensure that patients receive accurate and safe guidance, which highlights the importance of regulatory frameworks, transparency, and ethical design in fostering trust and accountability in AI-driven healthcare. Consistent with this, clinicians emphasised the need for clear medicolegal guidance and explicit accountability structures before integrating AI into decision-making [34,35].

#### 3.2.4. Organisational Factors

Beyond individual and ethical considerations, the success of AI adoption is also associated with the organisational environment in which the technology is used. Organisational support is therefore a critical enabler of AI adoption in primary care, as this support impacts everything from implementation logistics to long-term sustainability. Leadership endorsement, alignment with national strategies, and integration into existing workflows are key facilitators identified within the literature. Bhambhwani et al. [46] demonstrated that when AI tools are effectively embedded into clinical workflows, they have the capacity to achieve high patient uptake and operational efficiencies. Similarly, Young et al. [41] highlighted the potential of EHR-integrated AI tools to support triage at a larger scale, suggesting that seamless integration with existing systems may enhance both clinician efficiency and patient outcomes.

Financial and institutional support also play a vital role, as Wewetzer et al. [37] emphasised the need for clear reimbursement policies and practice-level support to encourage adoption, particularly in resource-constrained settings. Yoon et al. [28] advocated for hybrid models that combine AI with human coaching, which may be more sustainable and acceptable to both patients and clinicians. Sangers et al. [29] highlighted the importance of medical college or professional society endorsement and of there being alignment with the various national guidelines, which may increase credibility and drive broader uptake [34]. Finally, da Silva Lima Roque et al. [42] stressed the need for training and usability support, particularly for non-specialist users in primary care [40,49]. Overall successful AI adoption requires technical readiness along with strategic alignment, institutional backing, seamless integration with existing systems, and ongoing support [34,35,39]. In addition, health workers and programme officers highlighted the importance of structured rollout plans and clear implementation strategies to ensure sustainability [33,40,49]. However, even with strong organisational foundations, the broader context in which AI is deployed, particularly geographic and cultural factors, can significantly shape the adoption and impact of AI.

#### 3.2.5. Geographical and Cultural Factors

Geographical and cultural contexts significantly influence the adoption and implementation of AI in primary care. In remote and underserved regions, such as rural and remote Australia [45] and Northern Ontario, Canada [46], AI tools were particularly valued for their ability to bridge access gaps. Mobile and autonomous AI systems enabled screening and diagnosis in areas with limited specialist availability, long travel distances, and harsh weather conditions. These tools were seen as essential for improving equity in healthcare delivery. However, readiness in these contexts varied. In countries such as Denmark and the UK, structured implementation frameworks and national health systems facilitated smoother integration of AI [31,35,44]. In contrast, studies from Germany and Australia highlighted challenges related to digital infrastructure, workforce shortages, and inconsistent funding [32,45].

However, cultural context and equity-related considerations also played a critical role in shaping safe and effective AI adoption. While Bhambhwani et al. [46] did not explore Indigenous Canadian perspectives, where only a small proportion of participants identified as Indigenous, this suggests a need for more inclusive outreach and culturally tailored implementation strategies. In similar contexts, such as rural Australia, Li et al. [45] emphasised the importance of culturally safe AI adoption among Aboriginal and Torres Strait Islander communities. This involved co-designing AI tools with local stakeholders, respecting community values, and addressing historical mistrust in healthcare systems. Cultural expectations around the patient-provider relationship also influence AI acceptance. In Singapore, Yoon et al. [28] found that patients valued the efficiency of app-based motivational interviewing but still preferred hybrid models that preserved human interaction, reflecting the broader cultural norms, where relational care and professional authority are highly valued in Singapore and other Asian regions. In India, many older adults relied on family members for digital navigation, reflecting caregiving norms that shape practical acceptance and sustained use of AI technologies [39]. Similarly, Chauhan et al. [40] found that community acceptance of screening, in India, depended heavily on family involvement and trust in known healthcare providers, shaping how AI-enabled tools were perceived and used across rural and community settings.

Importantly, these geographical and cultural considerations should not be viewed as secondary contextual factors but as core implementation requirements for AI adoption in primary care. Algorithmic bias, driven in part by the underrepresentation of diverse populations in training datasets, may contribute to reduced accuracy and inequitable performance, particularly for Indigenous, rural, and culturally and linguistically diverse populations [45,47]. Language barriers and varying levels of digital literacy may further limit equitable access and effective use of AI-enabled tools, especially in chronic disease management, where patient engagement and self-management are central [39,40,46]. As such, culturally safe co-design, involving patients, communities, and local healthcare providers, is essential to ensure that AI systems are acceptable, accessible, and aligned with community values [45]. Embedding these considerations early in the design and implementation process, rather than addressing them retrospectively, is critical for achieving equitable, safe, and sustainable AI adoption in primary care.

## 4. Discussion

This review identified five interrelated drivers that shape AI adoption in primary care chronic disease management: technical, human, legal and ethical, organisational, and geographical and cultural factors. Overall, the findings indicate that although AI adoption is perceived as promising for improving access, efficiency, and decision support in chronic disease care, uptake is constrained by implementation challenges that extend beyond algorithm performance. Across studies, successful adoption was most likely when AI tools were built on fit-for-purpose data, integrated smoothly into existing systems and workflows, governed by clear accountability and privacy safeguards, and introduced in ways that preserved relational care and addressed local contextual needs. These findings align with broader reviews indicating that trust, governance, and system readiness are central determinants of AI adoption in healthcare [19,22], and they reinforce calls to evaluate AI using broader metrics that capture organisational and social impacts rather than technical accuracy alone [23]. It is important to note, however, that not all included studies directly evaluated AI adoption or implementation outcomes. While qualitative, implementation, usability, and feasibility studies provided empirical evidence regarding adoption determinants, some validation, prediction, and observational studies contributed implementation-relevant insights relating to data quality, external validation, workflow integration, interoperability, transparency, and deployment readiness. These factors were interpreted as potentially influential drivers of adoption rather than as directly measured determinants or predictors of adoption behaviour.

Technical barriers are often foundational, particularly limitations in data availability, data quality, dataset representativeness, interoperability, and transparency. Included studies highlighted that missing or incomplete clinical inputs undermined the robustness and validity of AI tools, while reliance on structured EHR data risked overlooking the contextual and longitudinal complexity central to chronic disease care [41]. Concerns about generalisability and bias due to non-diverse training data were also recurrent, echoing the wider literature on algorithmic bias and the risks of inequitable performance for underrepresented groups [19]. Interoperability and integration challenges, including high device costs and poor compatibility with existing systems, are consistent with previously reported technological and infrastructural barriers to AI adoption across healthcare [21]. Collectively, these findings indicate that technical readiness is not merely a ‘back-end’ issue but a practical determinant of trust, feasibility, and scalability in a real-world primary care context.

Human factors shape both acceptance and sustained use, with clinician trust, perceived workload implications, and training needs emerging as major influences. Concerns about job displacement, time burden, and disruption to clinical workflows reflect common themes in implementation research, where perceived usefulness and ease of use are critical to adoption, especially in time-constrained primary care consultations. In the updated CFIR mapping, these findings aligned principally with the Individuals domain, particularly Capability, Opportunity, and Motivation, while workload, training, and workflow issues also intersected with Inner Setting constructs such as Compatibility and Access to Knowledge and Information [22,25]. Patient-facing studies similarly highlight trust, autonomy, and digital literacy, with preferences for empathetic human interaction supporting the view that AI tools are more acceptable when they complement rather than replace clinician-patient relationships. This aligns with broader health AI adoption literature, which emphasised trustworthiness, transparency, and user-centred design as prerequisites for uptake [19].

Legal and ethical challenges were repeatedly linked to uncertainty around accountability, privacy, and fairness, particularly regarding responsibility when AI produces incorrect recommendations. These concerns are widely documented in the AI adoption literature and reinforce the centrality of governance arrangements, regulatory clarity, and ethical oversight for responsible implementation [19]. Studies also raised concerns about transparency and explainability as conditions for informed decision-making and patient autonomy, consistent with growing calls for explainable AI in clinical contexts [50]. Potential conflicts of interest and the need for independent validation further highlight the importance of rigorous evaluation and transparency in reporting. These findings support arguments that evaluation must extend beyond technical accuracy to include ethical, legal, and social implications in real-world routine practice [23].

Organisational factors emerged as key enablers of adoption and sustainability, particularly leadership endorsement, workflow integration, training support, professional-body alignment, and reimbursement structures. They align closely with implementation science evidence that organisational readiness, resources, and implementation strategies shape whether innovations become embedded in routine care [25]. It is suggested that when AI tools are integrated into existing systems and supported by institutional policies and funding mechanisms, they may achieve higher uptake and operational efficiency. This aligns with broader adoption reviews highlighting governance, resourcing, and professional support as facilitators [19], and with CFIR constructs relating Compatibility, Available Resources, Financing, and the engagement of High-Level and Mid-Level Leaders, which collectively influence the extent to which innovations can be integrated into existing workflows and sustained within organisational settings [22]. Additionally, organisational facilitators were linked to acceptability, as hybrid models combining AI with human support were described as more sustainable and better aligned with the relational and longitudinal care models of primary care [28]. These findings further align with CFIR constructs associated with Access to Knowledge and Information, Planning, and Reflecting and Evaluating, highlighting the importance of implementation support, training, and ongoing organisational learning in sustaining AI adoption [22].

Geographical and cultural factors shape adoption through differences in infrastructure, workforce capacity, health system organisation, and sociocultural expectations for care. Studies from rural and remote settings suggest that AI can play a compensatory role by supporting screening and triage in settings where specialist access is limited, potentially contributing to equity goals [45,46,48]. However, these benefits were contingent on local readiness and infrastructure, echoing broader evidence that uneven digital capacity can widen rather than reduce disparities if implementation is not context-sensitive [19]. Cultural considerations, including the need for culturally safe and co-designed approaches for Indigenous communities, further reinforce that AI adoption is not value-neutral. Preferences for hybrid models that preserve human interaction indicate that relational care remains central to acceptability and suggest that implementation strategies should be tailored to local cultural norms and expectations. Overall, these findings suggest that AI adoption in primary care is a socio-technical and context-dependent process that requires alignment across technical systems, human factors, organisational structures, and cultural contexts.

Recent advances in generative artificial intelligence, particularly large language models (LLMs) and retrieval-augmented generation (RAG) systems, further extend the landscape of AI adoption in primary care chronic disease management [51]. Unlike earlier AI applications focused primarily on prediction or classification, LLM-based systems enable more interactive and context-aware functions, including patient education, clinical documentation support, summarising electronic health records, and evidence-informed decision support [52]. These capabilities are particularly relevant for chronic disease management, where longitudinal care, multimorbidity, and ongoing patient self-management require continuous synthesis of complex clinical and contextual information [51,52].

Emerging literature highlights both opportunities and challenges associated with these technologies. For example, generative AI tools such as ChatGPT 5.6 (San Francisco, USA) have demonstrated the potential to enhance clinician efficiency and patient engagement through conversational interfaces and personalised health information delivery [52]. Similarly, RAG-based approaches aim to improve the reliability of AI outputs by grounding responses in curated and up-to-date clinical evidence, addressing key concerns around hallucination and misinformation [51]. However, these systems also introduce new implementation challenges that align with and extend the drivers identified in this review, including issues of trust, explainability, transparency, data governance, and workflow integration. Concerns regarding hallucination risk, variable accuracy, and medico-legal accountability further reinforce the need for robust validation, human oversight, and evidence-grounded design.

From an implementation perspective, the integration of LLMs and RAG pipelines into primary care settings underscores the importance of socio-technical alignment [51,52]. These systems must not only demonstrate technical performance but also fit within time-constrained consultations, support clinician decision-making without increasing cognitive burden, and maintain patient trust through transparent and safe operation. In this way, recent developments in generative AI reinforce the central findings of this review that successful AI adoption in chronic disease management depends on coordinated attention to technical, human, organisational, and ethical factors, while also introducing new considerations related to evidence grounding, conversational interaction, and the safe deployment of generative models in clinical practice [51,52].

### 4.1. Implications and Recommendations

While the preceding findings describe reported barriers and facilitators from the included studies, the following implications represent interpretive recommendations derived from the synthesis of these findings. Based on the findings of this review, successful AI adoption for chronic disease management in primary care may be supported by treating implementation as a socio-technical process, rather than a purely technical deployment. For practice, uptake and sustainability may be strengthened by embedding AI tools into routine workflows, providing targeted training and digital literacy support for clinicians and patients, and adopting hybrid models that preserve relational care and ensure appropriate human oversight or review [28,29,31]. Preference should be given to tools that minimise additional documentation burden, integrate with existing practice software, and rely primarily on routinely collected data to fit time-constrained primary care consultations. For policy and governance, clear regulatory and organisational arrangements are needed to address accountability, privacy, equity, and safety, including expectations for transparency, independent validation, and ongoing post-implementation monitoring in real-world settings [31,46]. Investment and reimbursement mechanisms also warrant attention, as device costs, integration expenses, and funding model uncertainty may constrain implementation in routine primary care [37,44]. Due to differences in infrastructure, workforce capacity, and cultural values, implementation strategies should also be tailored to local contexts, particularly in rural, remote, and underserved settings and populations with cultural and linguistic diversity [32,45].

These findings also have important implications for nursing and primary care practice. Nurses play a critical role in supporting the integration of AI into routine care through patient education, digital literacy support, and the facilitation of self-management for chronic conditions. In primary care settings, nurses are often central to care coordination, monitoring, and follow-up and may be well positioned to support AI-assisted screening, triage, and remote monitoring activities. In addition, nurses contribute to maintaining patient-centred care by providing clinical oversight, interpreting AI-generated outputs in context, and ensuring that care decisions remain safe, ethical, and aligned with individual patient needs. As such, successful AI adoption in primary care is likely to depend on actively engaging nursing roles in co-design, workflow integration, and ongoing evaluation of AI-enabled models of care.

### 4.2. Limitations

Several limitations are present within the scoping review. Only studies published in English were included, which may have potentially excluded relevant evidence from non-English-speaking contexts and introduced language bias.

Further, the search strategy was limited to peer-reviewed literature, and grey literature sources such as policy reports, organisational documents, and technical evaluations were not included. In addition, almost half of the included studies focused on diabetic retinopathy screening, meaning that many identified implementation drivers were derived from screening-oriented AI applications rather than broader chronic disease management interventions. Consequently, transferability of findings to other chronic disease contexts such as multimorbidity management, medication optimisation, remote monitoring, self-management support, and care coordination should be interpreted cautiously.

A further limitation is that several included studies were validation, prediction, feasibility, or observational investigations rather than studies explicitly designed to evaluate adoption or implementation outcomes. Consequently, some factors identified in the synthesis represent implementation-relevant considerations derived from empirical evaluations of system performance, usability, or deployment requirements, whereas others were directly reported adoption determinants. The findings should therefore be interpreted as representing the range of factors likely to influence AI adoption in primary care rather than definitive evidence of their relative contribution to adoption behaviour.

In addition, methodological quality appraisal was not conducted, consistent with scoping review guidance and the review objectives, whereby the focus is on mapping the breadth and nature of available evidence rather than evaluating study rigour. However, the absence of quality appraisal has implications for the interpretation of findings. The synthesis reflects reported drivers of AI adoption across the literature but does not distinguish between higher- and lower-quality studies or weight findings according to methodological strength. Consequently, it is possible that evidence from weaker or exploratory studies may have influenced the thematic synthesis. The findings should therefore be interpreted as indicative of the range and nature of implementation factors rather than as definitive evidence of their relative importance or effect.

Additional limitations include the intentional focus on primary care settings and chronic disease management, which may reduce transferability to specialist, acute, or broader public health contexts where AI adoption dynamics may differ. Furthermore, heterogeneity in study designs, AI applications, and outcome reporting limited comparability across studies and may have constrained the depth of interpretation. Future research employing more rigorous study designs and formal evaluation of implementation outcomes is needed to strengthen the evidence base in this area.

## 5. Conclusions

A range of key drivers that influence the adoption of AI to support chronic disease management in primary care were identified across multiple levels, including technical, human, legal and ethical, organisational, and broader geographical and cultural factors. Given the multi-level nature of these drivers, coordinated strategies are likely required across health systems, organisations, and individuals to support safe and sustainable AI adoption. These strategies may include strengthening data governance and interoperability standards, establishing clear regulatory and accountability frameworks, investing in workforce capability and digital literacy, supporting workflow-aligned implementation and hybrid models with appropriate human oversight, and providing resourcing and reimbursement mechanisms that enable routine use in primary care. Implementation approaches should also be tailored to local infrastructure and community values, including culturally safe co-design in underserved and diverse populations, to support trust and equity. More rigorous empirical studies are also needed in the future to examine how these strategies perform in real-world primary care and to evaluate outcomes beyond technical accuracy, including acceptability, safety, workload, equity, and longer-term organisational impacts.

## Figures and Tables

**Table 1:** Overview of each of the identified studies.

Author (Year)	Country	Study Design	AI Type	Chronic Disease Focus	Primary Care Context	Key Adoption Driver
Abuzour et al. [33]	UK	Qualitative	Decision support tool	Multimorbidity/polypharmacy	General practice	Workflow integration, usability
Alanzi et al. [38]	Saudi Arabia	Cross-sectional	AI assistants	General family medicine	Primary care centres	Trust, facilitating conditions
Bhambhwani et al. [46]	Canada	Prospective cohort feasibility	Screening AI	Diabetic retinopathy	Primary care clinic	Access, patient satisfaction
Bora et al. [43]	USA/ Thailand	Retrospective Validation	Deep learning prediction	Diabetic retinopathy	Screening/clinical data use	Predictive accuracy, data integration
Chauhan et al. [40]	India	Cross-sectional study	Screening AI	Diabetic retinopathy	Community and facility care	Access, usability, trust
Chauhan et al. [49]	India	Observational	Screening AI	Diabetic retinopathy	Community-based screening	Point-of-care access, scalability
Cooper et al. [34]	UK	Qualitative	Decision support	Multimorbidity	General practice	Trust, clinician acceptance
d’Elia et al. [35]	UK	Qualitative	Decision support	Multimorbidity	Primary care prescribing	Integration, medico-legal clarity
da Silva Lima Roque et al. [42]	Brazil	Validation	Chatbot	Wound care	Patient/self-care support	Usability, accessibility
Doe et al. [30]	UK	Qualitative	AI-assisted interpretation	Chronic respiratory disease	Primary care spirometry	Quality assurance, workforce support
Held et al. [32]	Germany	Qualitative	Screening AI	Diabetic retinopathy	Primary care clinics	Training, organisational readiness
Ipp et al. [36]	USA	Prospective multicentre cross-sectional	Screening AI	Diabetic retinopathy	Primary and eye care	Diagnostic accuracy, ease of use
Krogh et al. [31]	Denmark	Qualitative feasibility	Screening AI	Diabetic retinopathy	General practice	Staff acceptability, workflow fit
Li et al. [45]	Australia	Prospective Implementation	Screening AI	Diabetic retinopathy	Remote primary care	Access, cultural acceptability
Nobes et al. [44]	UK	Implementation	Expert system	Liver disease	Primary care testing	Integration, cost-effectiveness
Nolan et al. [48]	USA	Prospective Observational	Screening AI	Diabetic retinopathy	Rural primary care	Access, patient acceptance
Rafi [39]	India	Cross-sectional mixed methods	AI-enabled telemedicine	Chronic disease management	Community and primary care	Digital empowerment, access
Romero-Brufau et al. [47]	USA	Pre-post Observational	Clinical decision support	Diabetes	Primary care clinics	Care coordination, team communication
Sangers et al. [29]	Netherlands	Qualitative	Diagnostic AI	Skin cancer	Primary care and dermatology	Diagnostic support, education
Wewetzer et al. [37]	Germany	Cross-sectional	Screening AI	Diabetic retinopathy	Primary care	Reimbursement, technical integration
Yoon et al. [28]	Singapore	Qualitative	AI app/chatbot	Diabetes self-management	Primary care clinics	Patient engagement, hybrid care
Young et al. [41]	USA	Retrospective case–controlled	Predictive AI/ML	Eye diseases (including diabetic retinopathy)	Electronic health record-supported primary care	Early risk prediction, scalability

**Table 2:** Matrix of drivers of AI adoption.

Author (Year)	Technical Factors	Human Factors	Legal and Ethical Factors	Organisational Factors	Geographical and Cultural Factors
Abuzour et al. [33]	Data; Validation	–	Liability	Workflow	–
Alanzi et al. [38]	Interoperability; Data	–	Privacy	Support	–
Bhambhwani et al. [46]	–	–	–	–	Rural access; indigenous underrepresented
Bora et al. [43]	Data Integration	–	–	–	–
Chauhan et al. [40]	Usability	Fear	–	Support	Rural; Cultural preference
Chauhan et al. [49]	Usability	Fear	–	Support	–
Cooper et al. [34]	–	Reliability	Liability	Support	–
d’Elia et al. [35]	–	Reliability	Liability	Workflow	Structured national implementation
da Silva Lima Roque et al. [42]	Data; usability	Interaction	–	Support	–
Doe et al. [30]	–	Fear; Workload	–	Support	–
Held et al. [32]	Data; Integration	Fear; Training	Liability	Support	Infrastructure; workforce challenges
Ipp et al. [36]	–	trust	–	–	–
Krogh et al. [31]	–	Workload; Trust	–	–	Structured national implementation
Li et al. [45]	Integration	–	–	–	Rural; Culturally safe AI for indigenous communities
Nobes et al. [44]	Data; Algorithm	Training	–	Support	Structured national implementation
Nolan et al. [48]	Integration	–	–	Workflow	–
Rafi [39]	Usability	Fear	Privacy	Workflow	Rural; Cultural preference
Romero-Brufau et al. [47]	Integration	–	–	Workflow	–
Sangers et al. [29]	Algorithm; Validation; Usability	Fear	Privacy; transparency; regulation	Support	–
Wewetzer et al. [37]	Usability	–	Liability	Reimbursement	–
Yoon et al. [28]	–	Literacy; Interaction	–	Support	Cultural preference for hybrid care models
Young et al. [41]	Data; Validation	–	–	Workflow; Support	–

Note: Terms used in Table 2 represent aggregated themes derived from the studies included. For example, “Data” refers to issues such as data quality, availability, completeness, and representativeness; “Fear” reflects concerns relating to trust, reliability, role displacement, and uncertainty about AI use; “Support” includes organisational, technical, or institutional enablers such as training, leadership, or infrastructure; and “Workflow” refers to integration into clinical processes, time constraints, and compatibility with routine care. Additional detail is provided in Section 3.2.1, Section 3.2.2, Section 3.2.3, Section 3.2.4 and Section 3.2.5.

**Table 3:** Mapping of empirically derived AI adoption domains to the updated CFIR.

Empirical Domain	Examples of Factors	Updated CFIR Domain(s)	Relevant CFIR Constructs
Technical	Data quality; representativeness; interoperability; usability; explainability	Innovation; Inner Setting	Innovation Evidence-Base; Innovation Design; Innovation Complexity; Information Technology Infrastructure; Compatibility
Human	Trust; workload; training/digital literacy; role concerns; hybrid human–AI acceptance	Individuals; Inner Setting	Capability; Opportunity; Motivation; Access to Knowledge and Information; Compatibility
Legal and ethical	Liability; privacy/security; fairness; regulation; validation	Outer Setting; Innovation; Inner Setting	Policies and Laws; Innovation Design; Innovation Evidence-Base; Human Equality-Centeredness
Organisational	Leadership; workflow fit; resources; reimbursement; implementation support	Inner Setting; Outer Setting; Implementation Process; Individuals	Compatibility; Available Resources; Financing; Planning; Reflecting and Evaluating; High-Level Leaders; Mid-Level Leaders
Geographical and cultural	Rural access; workforce; cultural safety/trust; co-design; language; digital access	Outer Setting; Individuals; Implementation Process	Local Conditions; Local Attitudes; Need/Capability/Opportunity/Motivation; Assessing Needs; Engaging; Adapting

Note: Mapping was based on the conceptual meaning of each factor rather than exact terminology. Where factors aligned with multiple CFIR constructs, a primary mapping was assigned according to best conceptual fit, with secondary alignments noted where relevant. Factors not fully represented within existing CFIR constructs were retained within the inductively derived domains rather than being forced into predefined categories.

## Data Availability

Dataset available on request from the authors.

## Supplementary Materials

The following supporting information can be downloaded at: https://www.mdpi.com/article/10.3390/nursrep16090315/s1, Table S1: Context and overview of each of the identified studies.

## Public Involvement Statement

No public involvement in any aspect of this research.

## Guidelines and Standards Statement

This manuscript was drafted against the PRISMA extension for Scoping Reviews (PRISMA-ScR) checklist.

## Appendix A

**Table A1:** MEDLINE (via PubMed).

No.	Query	Results
#1	(“Primary Health Care”[Mesh] OR “Family Practice”[Mesh] OR “primary care”[Title/Abstract] OR “primary healthcare”[Title/Abstract] OR “general pract”[Title/Abstract] OR “practice nurs”[Title/Abstract]* OR “community health”[Title/Abstract] OR “family medicine”[Title/Abstract])	200,790
#2	#1 AND (“Artificial Intelligence”[Mesh] OR “Machine Learning”[Mesh] OR “artificial intelligen”[Title/Abstract] OR AI[Title/Abstract] OR “machine learning”[Title/Abstract] OR “deep learning”[Title/Abstract] OR “cognitive computing”[Title/Abstract] OR “neural network”[Title/Abstract] OR “natural language processing”[Title/Abstract] OR chatbot[Title/Abstract] OR “conversational agent”[Title/Abstract] OR ”expert system”[Title/Abstract]**)	2826
#3	#2 AND (adoption[Title/Abstract] OR implementation[Title/Abstract] OR barrier[Title/Abstract] OR hinder[Title/Abstract] OR obstacle[Title/Abstract] OR challenge[Title/Abstract]* OR difficult[Title/Abstract] OR enabl[Title/Abstract] OR facilitat[Title/Abstract])	1371
#4	#3 AND (“Chronic Disease”[Mesh] OR “chronic disease”[Title/Abstract] OR “disease, chronic”[Title/Abstract] OR “diseases, chronic”[Title/Abstract] OR “chronic condition”[Title/Abstract] OR “chronic illnesses”[Title/Abstract] OR chronic*[Title/Abstract] OR “Long-Term Care”[Title/Abstract] OR “Long-term”[Title/Abstract] OR longterm[Title/Abstract] OR “Non-communicable*”[Title/Abstract] OR “chronic illness”[Title/Abstract] OR “chronically ill”[Title/Abstract] OR “chronic patient”[Title/Abstract] OR “heart attack”[Title/Abstract] OR “cardiovascular disease”[Title/Abstract] OR neoplasm*[Title/Abstract] OR tumo*[Title/Abstract] OR neoplasia[Title/Abstract] OR cance*[Title/Abstract] OR carcinom*[Title/Abstract] OR malignant[Title/Abstract] OR oncolog*[Title/Abstract] OR “chronic obstructive pulmonary disease”[Title/Abstract] OR COPD[Title/Abstract] OR “chronic pulmonary disease”[Title/Abstract] OR “chronic respiratory diseases”[Title/Abstract] OR asthma[Title/Abstract] OR diabetes[Title/Abstract] OR “musculoskeletal conditions”[Title/Abstract] OR “Chronic Pain”[Title/Abstract])	473

2010–2026 = 473 (Title/Abstract and MeSH fields).

**Table A2:** IEEE Xplore.

No.	Query	Results
#1	(“Primary Care” or “Primary Healthcare” or “General Pract*” or “Practice Nurs*” or “Community Health*” OR “family medicine”)	227
#2	#1 AND (“adoption” OR “implementation” OR “barrier*” or “hinder*” or “obstacl*” or “challeng*” or “difficult*” or “enabl*” or “empower” or “facilitat*”)	21
#3	#2 AND (“chronic disease” OR “disease, chronic” OR “diseases, chronic” OR “Chronic condition” OR “chronic illnesses” OR “chronic*” OR “Long-Term Care” OR “Long-term*” OR “Longterm* “OR “Non-communicable*” OR “chronic illness” OR “chronically ill” OR “chronic patient” OR “heart attack” OR “cardiovascular disease” OR “neoplasm*” OR “tumo*” OR “neoplasia”OR “cance*” OR “carcinom*”OR “malignant” OR “Oncolog*” OR “chronic obstructive pulmonary disease” OR “COPD” OR “chronic pulmonary disease” OR “chronic respiratory diseases” OR “asthma” OR “diabetes” OR “musculoskeletal conditions” OR “Chronic Pain”)	5
#4	#3 AND (“Intelligence, Artificial”, OR “Machine Learning” OR “artificial intelligen*” OR “AI” OR “big learning” OR “deep learning” OR “automation” OR “cognitive computing” OR “neural networks” OR “intellig* computing” OR “natural language processing” OR “chatbot*” OR “chat robot” OR “conversational agent” OR “expert system” OR “Robotics”)	0

Limits applied: English language; 2010–2026.

**Table A3:** Scopus.

No.	Query	Results
#1	TITLE-ABS (“Primary Care” or “Primary Healthcare” or “General Pract*” or “Practice Nurs*” or “Community Health*” OR “family medicine”)	295,370
#2	#1 AND TITLE-ABS (“Intelligence, Artificial”, OR “Machine Learning” OR “artificial intelligen*” OR “AI” OR “big learning” OR “deep learning” OR “automation” OR “cognitive computing” OR “neural networks” OR “intellig* computing” OR “natural language processing” OR “chatbot*” OR “chat robot” OR “conversational agent” OR “expert system” OR “Robotics”)	5587
#3	#2 AND TITLE-ABS (“adoption” OR “implementation” OR “barrier*” or “hinder*” or “obstacl*” or “challeng*” or “difficult*” or “enabl*” or “empower” or “facilitat*”)	2458
#4	#3 AND TITLE-ABS (“chronic disease” OR “disease, chronic” OR “diseases, chronic” OR “Chronic condition” OR “chronic illnesses” OR “chronic*” OR “Long-Term Care” OR “Long-term*” OR “Longterm* “OR “Non-communicable*” OR “chronic illness” OR “chronically ill” OR “chronic patient” OR “heart attack” OR “cardiovascular disease” OR “neoplasm*” OR “tumo*” OR “neoplasia”OR “cance*” OR “carcinom*”OR “malignant” OR “Oncolog*” OR “chronic obstructive pulmonary disease” OR “COPD” OR “chronic pulmonary disease” OR “chronic respiratory diseases” OR “asthma” OR “diabetes” OR “musculoskeletal conditions” OR “Chronic Pain”)	665

2010–2026 = 665 (TITLE-ABS fields only).

**Table A4:** Cochrane Library.

No.	Query	Results
#1	(“Primary Care” or “Primary Healthcare” or “General Pract*” or “Practice Nurs*” or “Community Health*” OR “family medicine”)	33,942
#2	#1 AND (“Intelligence, Artificial”, OR “Machine Learning” OR “artificial intelligen*” OR “AI” OR “big learning” OR “deep learning” OR “automation” OR “cognitive computing” OR “neural networks” OR “intellig* computing” OR “natural language processing” OR “chatbot*” OR “chat robot” OR “conversational agent” OR “expert system” OR “Robotics”)	695
#3	#2 AND (“adoption” OR “implementation” OR “barrier*” or “hinder*” or “obstacl*” or “challeng*” or “difficult*” or “enabl*” or “empower” or “facilitat*”)	24
#4	#3 AND (“chronic disease” OR “disease, chronic” OR “diseases, chronic” OR “Chronic condition” OR “chronic illnesses” OR “chronic*” OR “Long-Term Care” OR “Long-term*” OR “Longterm* “OR “Non-communicable*” OR “chronic illness” OR “chronically ill” OR “chronic patient” OR “heart attack” OR “cardiovascular disease” OR “neoplasm*” OR “tumo*” OR “neoplasia”OR “cance*” OR “carcinom*”OR “malignant” OR “Oncolog*” OR “chronic obstructive pulmonary disease” OR “COPD” OR “chronic pulmonary disease” OR “chronic respiratory diseases” OR “asthma” OR “diabetes” OR “musculoskeletal conditions” OR “Chronic Pain”)	0

Limits applied: English language; 2010–2026; Title, Abstract and Keyword fields.

**Table A5:** EMBASE.

No.	Query	Results
#1	‘primary care’/exp OR ‘primary care’ OR (primary AND (‘care’/exp OR care)) OR ‘primary healthcare’:ti OR ‘general pract*’:ti OR ‘practice nurse’:ti OR ‘community health*’:ti OR ‘family medicine’:ti	1,107,691
#2	‘intelligence, artificial’ OR ((‘intelligence,’/exp OR intelligence,) AND artificial) OR ‘machine learning’:ti OR ‘artificial intelligen*’:ti OR ai:ti OR ‘big learning’:ti OR ‘deep learning’:ti OR automation:ti OR ‘cognitive computing’:ti OR ‘neural networks’:ti OR ‘intellig* computing’:ti OR ‘natural language processing’:ti OR chatbot*:ti OR ‘chat robot’:ti OR ‘conversational agent’:ti OR ‘expert system’:ti OR robotics:ti	348,779
#3	‘adoption’/exp OR adoption OR implementation:ti OR barrier*:ti OR hinder*:ti OR obstacl*:ti OR challeng*:ti OR difficult*:ti OR enabl*:ti OR empower:ti OR facilitat*:ti	742,153
#4	‘chronic disease’/exp OR ‘chronic disease’ OR ‘disease chronic’:ti OR ‘chronic condition’:ti OR ‘chronic illnesses’:ti OR chronic*:ti OR ‘long term care’:ti OR ‘long term*’:ti OR longterm*:ti OR ‘non communicable’:ti OR ‘chronic illness’:ti OR ‘chronically ill’:ti OR ‘chronic patient’:ti OR ‘heart attack’:ti OR ‘cardiovascular disease’:ti OR neoplasm*:ti OR tumo*:ti OR neoplasia:ti OR cance*:ti OR carcinom*:ti OR malignant:ti OR oncolog*:ti OR ‘chronic obstructive pulmonary disease’:ti OR copd:ti OR ‘chronic pulmonary disease’:ti OR ‘chronic respiratory diseases’:ti OR asthma:ti OR diabetes:ti OR ‘musculoskeletal conditions’:ti OR ‘chronic pain’:ti	5,809,955
#5	#1AND #2 AND #3 AND #4	151

2010–2026 = 151 (Emtree terms and Title fields).

**Table A6:** PsycINFO.

No.	Query	Results
#1	TI,AB (“Primary Care” or “Primary Healthcare” or “General Pract*” or “Practice Nurs*” or “Community Health*” OR “family medicine”)	153,282
#2	#1 AND TI,AB (“Intelligence, Artificial”, OR “Machine Learning” OR “artificial intelligen*” OR “AI” OR “big learning” OR “deep learning” OR “automation” OR “cognitive computing” OR “neural networks” OR “intellig* computing” OR “natural language processing” OR “chatbot*” OR “chat robot” OR “conversational agent” OR “expert system” OR “Robotics”)	1191
#3	#2 AND TI,AB (“adoption” OR “implementation” OR “barrier*” or “hinder*” or “obstacl*” or “challeng*” or “difficult*” or “enabl*” or “empower” or “facilitat*”)	454
#4	#3 AND TI,AB (“chronic disease” OR “disease, chronic” OR “diseases, chronic” OR “Chronic condition” OR “chronic illnesses” OR “chronic*” OR “Long-Term Care” OR “Long-term*” OR “Longterm* “OR “Non-communicable*” OR “chronic illness” OR “chronically ill” OR “chronic patient” OR “heart attack” OR “cardiovascular disease” OR “neoplasm*” OR “tumo*” OR “neoplasia”OR “cance*” OR “carcinom*”OR “malignant” OR “Oncolog*” OR “chronic obstructive pulmonary disease” OR “COPD” OR “chronic pulmonary disease” OR “chronic respiratory diseases” OR “asthma” OR “diabetes” OR “musculoskeletal conditions” OR “Chronic Pain”)	97

2010–2026 = 97 (Title and Abstract fields).

**Table A7:** CINAHL.

No.	Query	Results
#1	(MH “Primary Health Care+” OR TI (“Primary Care” OR “Primary Healthcare” OR “General Pract*” OR “Practice Nurs*” OR “Community Health*” OR “family medicine”) OR AB (“Primary Care” OR “Primary Healthcare” OR “General Pract*” OR “Practice Nurs*” OR “Community Health*” OR “family medicine”))	109,030
#2	#1 AND (MH “Artificial Intelligence+” OR TI (“Artificial Intelligence” OR “Machine Learning” OR “artificial intelligen*” OR “AI” OR “big learning” OR “deep learning” OR “automation” OR “cognitive computing” OR “neural networks” OR “intellig* computing” OR “natural language processing” OR “chatbot*” OR “chat robot” OR “conversational agent” OR “expert system” OR “Robotics”) OR AB (“Artificial Intelligence” OR “Machine Learning” OR “artificial intelligen*” OR “AI” OR “big learning” OR “deep learning” OR “automation” OR “cognitive computing” OR “neural networks” OR “intellig* computing” OR “natural language processing” OR “chatbot*” OR “chat robot” OR “conversational agent” OR “expert system” OR “Robotics”))	790
#3	#2 AND (TI (“adoption” OR “implementation” OR “barrier*” OR “hinder*” OR “obstacl*” OR “challeng*” OR “difficult*” OR “enabl*” OR “empower” OR “facilitat*”) OR AB (“adoption” OR “implementation” OR “barrier*” OR “hinder*” OR “obstacl*” OR “challeng*” OR “difficult*” OR “enabl*” OR “empower” OR “facilitat*”))	322
#4	#3 AND (MH “Chronic Disease+” OR TI (“chronic disease” OR “disease, chronic” OR “diseases, chronic” OR “chronic condition” OR “chronic illnesses” OR “chronic*” OR “Long-Term Care” OR “Long-term*” OR “Longterm*” OR “Non-communicable*” OR “chronic illness” OR “chronically ill” OR “chronic patient” OR “heart attack” OR “cardiovascular disease” OR “neoplasm*” OR “tumo*” OR “neoplasia” OR “cance*” OR “carcinom*” OR “malignant” OR “Oncolog*” OR “chronic obstructive pulmonary disease” OR “COPD” OR “chronic pulmonary disease” OR “chronic respiratory diseases” OR “asthma” OR “diabetes” OR “musculoskeletal conditions” OR “Chronic Pain”) OR AB (“chronic disease” OR “disease, chronic” OR “diseases, chronic” OR “chronic condition” OR “chronic illnesses” OR “chronic*” OR “Long-Term Care” OR “Long-term*” OR “Longterm*” OR “Non-communicable*” OR “chronic illness” OR “chronically ill” OR “chronic patient” OR “heart attack” OR “cardiovascular disease” OR “neoplasm*” OR “tumo*” OR “neoplasia” OR “cance*” OR “carcinom*” OR “malignant” OR “Oncolog*” OR “chronic obstructive pulmonary disease” OR “COPD” OR “chronic pulmonary disease” OR “chronic respiratory diseases” OR “asthma” OR “diabetes” OR “musculoskeletal conditions” OR “Chronic Pain”))	116

2010–2026 = 116 (Subject Headings, Title and Abstract fields).
