# Supplementary material for: Drivers of Artificial Intelligence (AI) Adoption in Supporting Chronic Disease Management in Primary Care: A Scoping Review
Source: Nurs Rep. 2026 Sep 2;16(9):315. doi: 10.3390/nursrep16090315 (PMC13609955; doi:10.3390/nursrep16090315)
Supplement: Supplementary file 1 [file nursrep-16-00315-s001.zip › nursrep-4476107-supplementary.pdf]

Table S1. Context and overview of each of the identified studies

Author (Year)	Country	Study Design	Population /Sample	Intervention /Exposure	Outcomes Measured	Key Findings	Negative factors of AI Adoption	Positive factors of AI Adoption	Limitations
Abuzour et al. [33]	United Kingdom	Qualitative study	21 HCPs (GPs, pharmacists, clinical pharmacologists, psychiatrists, policy maker); 13 patients with multimorbidity/frailty	Exploration of requirements for an AI assisted tool (DynAIRx) to support structured medication reviews	AI potential, workflow integration, visualization, tool functionality, and implementation barriers and facilitators	AI may streamline structured medication reviews by prioritising high risk patients, visualising medical journeys, identifying prescribing gaps, and supporting shared decision making. Success depends on seamless integration, usability, and explainability	Concerns regarding workflow disruption, pop up fatigue, skepticism of predictive accuracy, need for evidence of improved outcomes, interoperability challenges, data governance, digital exclusion	User centred design; timeline visualisations; colour coded labs; targeted alerts; automated clinical calculators; capacity aware patient prioritisation; integration; improved patient engagement	Participant bias towards tech interested clinicians; limited generalisability to broader patient population; findings based on perceptions rather than real world tool use, no patient demographic data, need for future prototype testing
Alanzi et al. [38]	Saudi Arabia	Cross-sectional study	157 family physicians from public hospitals and primary care centres	Use of AI-enabled assistants in family medicine	Factors influencing AI acceptance (e.g., performance expectancy, effort expectancy, social influence, facilitating conditions, trust, privacy risk, ethical concerns,	78.9% used AI assistants; effort expectancy and facilitating conditions were strong positive influences; privacy risk negatively correlated with all factors; younger physicians	Privacy risk, ethical concerns, lack of regulatory support, poor interoperability standards	Access to data, increased computing power, telemedicine, effort expectancy, facilitating conditions	Limited to family physicians; cross-sectional design; self-reported data; potential selection bias; no exploration of mediating/moderating effects

					personal innovativeness)	showed more positive attitudes			
Bhambhwani et al. [46]	Canada	Prospective cohort feasibility study	202 patients with diabetes in a primary care clinic	Autonomous AI-based Diabetic Retinopathy screening using EyeArt system	Feasibility, patient satisfaction, referral adherence	93.6% exam completion rate; 22.2% had referable for Diabetic Retinopathy; 76.2% referral adherence; high satisfaction (mean 4.8/5)	Minor barriers included small pupils, refusal of dilation, and limited diversity in sample	High patient satisfaction, no need for dilation, point-of-care results, reduced need for separate appointments, increased access in underserved areas	Underrepresentation of Indigenous and non-Caucasian populations; limited to one urban setting; excludes patients without primary care access
Bora et al. [43]	USA and Thailand	Retrospective validation study	575,431 eyes for development; 7976 eyes in internal validation (USA); 4762 eyes in external validation (Thailand)	Deep-learning system using fundus photographs to predict risk of developing diabetic retinopathy within 2 years	Predictive accuracy, comparison with traditional risk factors, calibration, and survival analysis	Deep-learning system outperformed traditional risk factors; prediction improved from 0.72 to 0.81 (internal) and from 0.62 to 0.71 (external) when combined with risk factors; high-risk patients had >80% chance of developing Diabetic Retinopathy	Limited availability of some risk factors (e.g., blood pressure); variability in grading; lack of device-specific performance data; potential misclassification of subtle Diabetic Retinopathy at baseline	Large, diverse datasets; integration with known risk factors; strong predictive performance; potential to personalise screening intervals and reduce burden	Retrospective design; single-eye analysis; limited generalisability to other populations; absence of Optical Coherence Tomography data; lack of prospective validation
Chauhan et al. [40]	India	Cross-sectional study	600 adults with diabetes (≥30 years). Qualitative stakeholders: people with diabetes,	Three diabetic retinopathy screening (DRS) models: (I) facility-based screening at	Referral recommendations and adherence within one month, reasons for non-	Referral adherence was low across all arms (13–17%) with no significant difference	Concerns about diagnostic trust (machine vs. ophthalmologist). Challenges with	Convenience and access for older/low mobility patients; quick offline AI results	Possible selection bias. 70% telephone follow up contact rate. Single district

			research optometrists, healthcare providers (ophthalmologists, optometrists, medical officers, and program officers	Health and Wellness Centres by non-ophthalmologists; (II) community-based AI at home using a smartphone fundus camera with offline AI; (III) standard care (counselling and referral to district hospital)	adherence, usability/acceptability across stakeholder groups	between models. Common barriers: lack of awareness, financial constraints, transport difficulties, competing family/work priorities, perceived good eye health.	image quality/lighting and maintaining test conditions in homes, training needs for graders, workflow and operational hurdles.	at point of care; potential to reduce unnecessary referrals and save specialist time. Strong stakeholder support for integrating AI/ telemedicine. Portable cameras enable outreach; alignment with national primary care initiatives	context limits generalisability; some variables captured only qualitatively; unable to systematically compare adherent vs. non adherent subgroups.
Chauhan et al. [49]	India	Observational study	600 adults with diabetes (≥30 years) across 30 villages; additional qualitative interviews with healthcare providers and frontline workers	Three diabetic retinopathy screening models: (I) facility-based screening at Health & Wellness Centres by non-ophthalmologists with human grading; (II) community-based AI assisted screening at home using a smartphone fundus camera with offline AI; (III) standard care (no screening,	Reach: identification, refusals, reasons; Implementation: delivery, consistency, content, challenges and adaptations; time and workflow data.	Refusal higher at facilities (40%) and lower in community screening (13%). Barriers included distance, transport, mobility limitations, perceived good eye health, existing medical conditions, and concerns about long term medications; technical issues.	Home based imaging challenges; ergonomic strain and positioning issues; operational hurdles and infrastructure instability; occasional community hesitancy toward outsiders; perceived needs-based care seeking rather than preventive screening	Offline AI enables point of care results and scalable outreach; community-based model improved access for older or less mobile patients; targeted adaptations and ergonomic improvements enhanced image quality and efficiency; integration with local stakeholders and ASHAs strengthened participation	No patient level data on decliners; limited socioeconomic data; single rural block context; health system factors not fully evaluated; some outcomes reported separately.

				counselling and referral)					
Cooper et al. [34]	United Kingdom	Qualitative study	20 healthcare professionals: GPs, geriatricians, nurses, pharmacists.	Exploration of how AI-enabled clinical decision support tools may support decision-making in the management of multiple long-term conditions. Discussions based on a clinical case study	Attitudes toward AI, expectations, concerns, perceived requirements; impact on clinical decision-making; perceived usability	Health care providers saw AI as potentially improving safety, quality, and efficiency of decision making. Expected AI to synthesise complex factors; however, emphasised the irreplaceable value of human judgement, empathy, and contextual reasoning; minimum requirements included transparency, integration with existing systems, time efficiency, and maintaining clinician autonomy	Concerns about loss of human connection; risk of inaccurate or dangerous recommendations; poor data quality and coding; medicolegal responsibility; potential to exacerbate digital exclusion; fear of depersonalisation; limited patient trust; lack of clarity in AI processes.	Potential for improved safety, efficiency, education, standardisation; ability to process complex multi-factorial cases; streamlining documentation and admin tasks; potential to support less experienced clinicians; adaptive tools could support diverse patient needs; seen as 'inevitable' and aligned with modern practice	Convenience sample from one UK region; absence of older health care provider perspectives; potential interviewer influence; participants possibly more AI-interested. Perceptions may not generalise beyond NHS context; attitudes shaped by hypothetical case rather than real tool use; limited exploration of patient-facing aspects
d'Elia et al. [35]	United Kingdom	Qualitative study	15 prescribing clinicians in NHS primary care (10 GPs including 2	Exposure to a mock-up of the OPTIMAL AI informed clinical	Usability, interpretation, practical implementation,	Participants found the tool generally clear, user friendly, valuable	Concerns about integration with existing electronic health records.	Seen as complementary to guidelines; provides new	Single-region sample limits generalisability; small. Artificial

			registrars, 3 nurse practitioners, 2 prescribing pharmacists)	decision support system for prescribing in multiple long-term conditions.	impact on shared decision making, attitudes toward AI	where guidelines are insufficient, and useful for revealing medication effects across comorbidities; could prompt clinicians to 'think outside the box' and challenge habitual prescribing; varied views on usefulness for shared decision making depending on patient preference and consultation context	Increased consultation time burden; medicolegal accountability and documentation; difficulty reconciling AI outputs with NICE guidelines; risk of misalignment between tool recommendations and clinical contraindications; lack of trust due to training data quality; resistance from clinicians valuing intuition and holistic judgement.	insights into medication impacts across conditions; helps standardise and optimise prescribing; acts like a 'colleague' or 'teacher'; supports lateral thinking; could assist in shared decision making for informed, engaged patients; appealing visual design; potential to improve safety and reduce prescribing error	nature of mock up may not reflect live use. Context specific to NHS primary care; limited ability to evaluate real world implementation
da Silva Lima Roque et al. [42]	Brazil	Validation study	7 wound care experts for validation; 17 users (patients, caregivers, nurses) for usability	BOTCURATIVO: a chatbot providing wound dressing guidance based on wound characteristics	Content validity and usability	High content validity (0.97); good usability (80.1); chatbot considered clear, relevant, and helpful for guiding wound care	Minor usability issues (e.g., font size, interface preferences); limited to text-based interaction; no clinical outcome data yet	Validated content, ease of use, visual aids, accessible via mobile, supports self-care and nurse guidance	Small sample size; limited generalisability; no voice or image recognition yet; early-stage prototype
Doe et al. [30]	United Kingdom	Qualitative study	28 stakeholders (clinicians and	Use of AI software in spirometry	Stakeholder perspectives on	Historic and pandemic-related	Scepticism about AI, fear of	AI can support quality assurance,	Limited to stakeholders with

			commissioners) involved in spirometry services across England	services post-COVID-19	spirometry provision, challenges, and the role of AI in quality and interpretation	challenges have led to inequities in spirometry access; stakeholders see potential for AI to support quality assurance and interpretation if well validated and trusted	deskilling, lack of understanding of AI capabilities, concerns over over-reliance on AI for diagnosis	reduce workload, improve interpretation accuracy, and help address workforce shortages	interest in spirometry; lacks patient perspectives; qualitative design limits generalisability
Held et al. [32]	Germany	Qualitative study	24 healthcare professionals (GPs, medical assistants, ophthalmologists)	AI-supported, smartphone-based device for diabetic retinopathy screening in primary care	Perceived barriers and facilitators to implementation of AI-assisted Diabetic Retinopathy screening	Identified 10 key determinants: personal attitude, organisation, time, financial factors, education, support, technical requirements, influence on profession, patient welfare	Uncertainty about AI accuracy, fear of deskilling, unclear legal liability, data security concerns, lack of training, technical integration issues, potential tension with ophthalmologists	Improved access in rural areas, delegation to trained staff, faster results, potential for cost-efficiency, stronger GP-patient relationship, support for overburdened systems	Limited to professionals in one region; potential selection bias; no patient perspectives; hypothetical implementation
Ipp et al. [36]	USA	Prospective multicentre cross-sectional diagnostic study	893 participants with diabetes (1786 eyes) from 15 primary and eye care centres	Use of EyeArt AI system for autonomous detection of more-than-mild and vision-threatening Diabetic Retinopathy	Sensitivity, specificity, imageability, and safety of AI system vs clinical reference standard	High sensitivity and specificity for both mtmDR (95.5%, 87.8%) and vtDR (97.0%, 90.1%); imageability 97.7%; effective without dilation in most cases; comparable performance in	Not explicitly discussed; potential concerns may include reliance on AI without physician oversight, and generalisability to other populations/settin gs	High diagnostic accuracy, ease of use by non-specialists, minimal need for dilation, potential to improve access and reduce burden on specialists	No use of Optical Coherence Tomography for Clinically Significant Macular Oedema confirmation; potential conflicts of interest; study funded by AI system developer

						primary and eye care settings			
Krogh et al. [31]	Denmark	Qualitative feasibility study	21 general practice staff from 12 clinics conducting AI-assisted Diabetic Retinopathy screening	AI-assisted diabetic retinopathy screening using a non-mydriatic fundus camera and SVML-based software (RetinaLyze)	Staff experiences and perspectives on feasibility, implementation, and implications of AI-assisted Diabetic Retinopathy screening in general practice	Screening is quick and easy; staff supported implementation; key needs included ergonomic setup, training, financial clarity, and defined referral pathways	Technical issues (e.g., lighting, connectivity), lack of Diabetic Retinopathy knowledge, unclear referral protocols, concerns about added workload, need for financial compensation	High acceptability, ease of use, improved access and attendance, reduced unnecessary referrals, staff trust in AI contingent on validity, potential to enhance diabetes care	Limited generalisability due to selection bias and overrepresentation of nurses; no theoretical framework applied
Li et al. [45]	Australia	Prospective, population-based implementation study	78 patients screened across 9 remote communities in Western Australia; 56.4% Aboriginal or Torres Strait Islander	Mobile Diabetic Retinopathy screening using AI-integrated retinal cameras operated by non-clinical staff	Screening uptake, patient acceptability, DR detection rates, image gradability	Eleven-fold increase in screening; 10.3% referable Diabetic Retinopathy; 96% patient approval of AI use; high feasibility and cultural acceptability	Equipment malfunction, internet issues, cost of mobile service, need for clinician supervision in some cases	High patient satisfaction, culturally co-designed model, real-time AI diagnosis, increased access in underserved areas, potential for primary care integration	Limited generalisability beyond mobile model; diagnostic accuracy of AI not validated in this study; sustainability concerns for mobile services
Nobes et al. [44]	United Kingdom (Scotland)	Implementation study	Over 11,000 screening tests performed in NHS Tayside	Intelligent Liver Function Testing (iLFT): an expert system algorithm for automated liver disease investigation and referral guidance	Uptake, diagnostic yield, referral rates, cost-effectiveness, system evolution	iLFT increased liver disease diagnosis by 43%, reduced unnecessary referrals, cost-effective, widely accepted by GPs;	Need robust IT infrastructure, training, data quality, and regulatory standards; potential over-referral from indeterminate	High GP satisfaction, automated testing, real-time decision support, adaptable algorithm, reduced care burden, cost savings	Limited to one area; some diagnostic thresholds under review; generalisability to other settings not yet tested

						74% of patients avoided referral	fibrosis scores; false positives in rare conditions		
Nolan et al. [48]	USA	Prospective observational study	102 patients and 13 primary care providers at a rural clinic	AI-assisted Diabetic Retinopathy screening using EyeArt in a primary care setting	Patient and provider experiences, perceived access, feasibility, and satisfaction	94% of patients rated the experience positively; 81% preferred future screenings at the clinic; 100% of providers agreed it improves access and screening rates	Limited availability of trained staff, scheduling challenges, initial workflow integration issues	High patient satisfaction, ease of access, no need for pupil dilation, point-of-care results, provider optimism about improved care	Small sample size, single site, no cost to patients may have biased responses, no comparison with specialist diagnosis
Rafi [39]	India	Cross sectional mixed methods	200 older adults (≥60 years); 20 elderly qualitative interviewees; 10 healthcare professionals	Use and perceptions of AI enabled telemedicine tools	Adoption rates; predictors of empowerment (digital literacy, family support); perceived benefits and challenges; systemic barriers	52% used at least one AI-enabled tool. Adoption higher among urban and more educated elders. Digital literacy and family support were strong predictors of empowerment.	Low digital literacy, high cost, lack of trust in AI decisions, dependence on younger family members, privacy/cybersecurity concerns; poor internet access; fear of reduced human contact; ethical concerns about data use; app usability issues	Increased access to healthcare; remote monitoring; improved disease management; improved medication adherence; reduced workload for healthcare professionals; convenience for mobility restricted elders; potential to enhance empowerment	Single-region sample limits generalisability. Cross-sectional design prevents causal inference. Self-reported measures subject to bias. Rapidly evolving AI landscape may outdate findings
Romero-Brufau et al. [47]	USA	Pre-post observational study	45 staff pre-implementation and 38 post-implementation	AI-based clinical decision support tool for diabetes management	Staff attitudes toward AI, perceived care coordination,	Improved perceptions of care coordination; decreased	Poorly tailored or irrelevant recommendations, high false-positive	Promoted team dialogue, increased familiarity with	Small sample, single setting, anonymous surveys prevented

			across 3 primary care clinics		usefulness of clinical decision support recommendations	excitement about AI post-implementation; only 14% would recommend the clinical decision support	rates, lack of integration into workflow, low perceived usefulness	AI, facilitated patient-centred discussions	paired analysis, no improvement in glycaemic control observed
Sangers et al. [29]	Netherlands	Qualitative study	33 participants (16 dermatologists, 17 GPs) across 6 focus groups	Perceptions of AI implementation in skin cancer care	Perceived benefits, barriers, and preconditions for AI use	AI seen as beneficial for improving diagnostic accuracy, care pathways, and education; potential to reduce unnecessary referrals and support GP training	Concerns about accuracy, lack of clinical context, algorithm transparency, health inequities, fear of replacement, time burden, privacy, and commercialization	Improved diagnostic confidence, educational value, potential for cost reduction, support from medical societies, clear validation and regulation, usability and accessibility	Views collected before actual implementation; limited to Dutch healthcare context; did not include perspectives on AI use by laypersons
Wewetzer et al. [37]	Germany	Cross-sectional	209 general practitioners	AI-based Diabetic Retinopathy screening in primary care	Perceived relevance of implementation factors	Financial factors such as costs and reimbursement and technical integration were most relevant; GPs saw potential for improved care and modern image	High acquisition and running costs, unclear reimbursement, liability concerns, lack of validated tools, low digital affinity	Perceived benefits to patient care, delegation to medical assistants, modern image, improved access, high device usability	Low response rate (10%), potential selection bias, no patient perspective included
Yoon et al. [28]	Singapore	Qualitative study	33 patients with type 2 diabetes mellitus from public primary care clinics	App-based motivational interviewing (MI) powered by AI for diabetes self-management	Acceptability of app-based MI, preferences for app features	Participants were generally open to app-based MI; valued convenience, anonymity, and	Concerns about loss of autonomy, lack of human touch, digital literacy challenges, and	Convenience, non-judgmental interaction, tailored feedback, visualisation tools, gamification, and	Small sample, limited cultural diversity, potential selection bias, mock-up app used

						personalisation; preferred hybrid model with human coaching	limited personalisation	hybrid model with human support	(not live), no long-term outcome data
Young et al. [41]	USA	Retrospective case-controlled study	1.49 million patients (743,039 with eye conditions, 743,039 matched controls)	AI/ML models applied to Electronic Health Record data to predict risk of five major eye diseases	Model accuracy, AUC, odds ratios for disease prediction	Average model accuracy ~80%; ORs for disease risk ranged from 2.4 (glaucoma) to 5.7 (type 1 non-proliferative diabetic retinopathy); models could support health care staff to triage	Data quality and completeness in Electronic Health Records; lack of doctor notes; potential over/under representation of diagnoses; need for validation	Uses only non-ophthalmic Electronic Health Record data; scalable and generalisable; could be integrated into health care staff workflows; supports early referral and triage	Retrospective design; no causal inference; no real-world deployment noted; lacks patient-level validation

Notes: AI: Artificial Intelligence; HCP: Healthcare Professional; GP: general practitioner; USA: United States of America; NICE: National Institute for Health and Care Excellence
